# Anaplastic Thyroid Carcinoma: Molecular Tools for Diagnosis and Therapy

**DOI:** 10.1155/2015/341725

**Published:** 2015-02-09

**Authors:** Ginesa Garcia-Rostan, Giovanni Tallini, Giuliana Salvatore

**Affiliations:** ^1^Institute of Molecular Biology and Genetics (IBGM), Valladolid University (Uva), Spanish Research Council (CSIC), C/ Sanz y Fores s/n, 47003 Valladolid, Spain; ^2^Scuola di Medicina e Chirurgia, Università di Bologna, Dipartimento di Medicina Specialistica, Diagnostica e Sperimentale, Anatomia Patologica, Ospedale Bellaria Via Altura 3, 40139 Bologna, Italy; ^3^Dipartimento di Scienze Motorie e del Benessere, Università Parthenope di Napoli, Via Medina 40, 80133 Naples, Italy; ^4^IRCSS SDN, Via E. Gianturco 113, 80143 Napoli, Italy

Anaplastic thyroid carcinoma (ATC) is one of the most lethal human malignancies, with a median overall survival of less than six months. While most of the genetic mutations occurring in papillary thyroid carcinoma (PTC) were recently discovered through an integrated genomic approach, molecular genetics of ATC is still in part unknown. ATC is refractory to conventional therapies, including surgery, chemotherapy, radiotherapy, and radioiodine therapy. New target agents are currently being evaluated in clinical trials. Under this frame, there is an urgent need to investigate the molecular biology and the therapeutic opportunities of this understudied, rare cancer.

The aim of this special issue is to gather a collection of papers focusing on molecular tools for diagnosis and therapy of anaplastic thyroid carcinoma. The issue contains eight papers including four review articles and four research papers.

The review article of C. S. Fuziwara and E. T. Kimura analyzes the role of miRNA in anaplastic thyroid carcinoma. miRNAs are a class of small noncoding RNAs that regulate posttranscriptional gene expression. Increasing evidences show that they may drive oncogenesis. The article underscores the families of miRNA that are deregulated in ATC, namely, the miR-200 family, miR-30 family, let-7 family, miR 17–92 cluster, miR-146a/b, and miR 221/222. Modulation of miRNA levels, using miRNA mimics or anti-miRs, may open new therapeutic options for ATC.

In the review article of M. Ragazzi et al. the focus is on the histopathology of ATC. The authors discussed the typical molecular features of ATC cells, with an elegant description of the main histological subtypes and the differential diagnostic criteria. The review included also an update of the molecular genetics of ATC.

The other two review articles included in the special issue focus on new proteins overexpressed in ATC, which may represent novel therapeutic targets, that is, Aurora kinases and transferrin receptor.

In detail, the work of E. Baldini et al. focuses on Aurora kinases in ATC. The review is a comprehensive update of the structure, expression, localization, and functions of Aurora proteins and their role in human cancers. Further, the review summarizes the findings, mainly made by the authors group, of Aurora kinases in thyroid cancers. Importantly, preclinical data indicate that Aurora kinase inhibitors may have a therapeutic potential for ATC treatment.

The review of R. Parenti et al. focuses on type 1 transferrin receptor (TfR1/CD71) in ATC. The TfR1/CD71 is a cell membrane glycoprotein involved in iron homeostasis and cell growth. The authors showed immunohistochemical data demonstrating the overexpression of TfR1/CD71 in ATC. They discuss the opportunities to target TfR1/CD71 by monoclonal or recombinant antibodies or transferrin-gallium-TfR1/CD71 molecular complexes or small interfering RNAs (siRNAs).

Four research articles are included in the special issue. Two of these articles are from the group of Sipetic. In the first research paper, V. Zivaljevic et al. analyze the risk factors for ATC. The study, done on 126 ATC patients, showed that independent risk factors for ATC are low education level, type B blood group, and goitre. A similar study, included in the special issue, from the same group of authors, addresses the importance of age in patient survival. The study demonstrates that the best prognosis for ATC is in patients younger than 50 years old.

In the research article by F. Baldan et al. the synergy between HDAC inhibitors and PARP inhibitors has been investigated in an ATC-derived cell line SW1736. The authors showed that both compounds synergize in activation of apoptosis and induction of thyroid-specific gene expression. Thus, this work suggests that the combined use of HDAC and PARP inhibitors may be a useful strategy for treatment of ATC.

Finally, W. Arancio and et al. conducted a competing endogenous RNA analysis to investigate the role of the stem cell factor SOX2 in ATC cell lines. The authors identified a functional network of SOX2 interactors including genes involved in the biogenesis of microRNAs (DICER1, RNASEN, and EIF2C2), in the control cell cycle (TP53 and CCND1), and in mitochondrial activity (COX8A).

In conclusion, within this special issue, we gather articles underlining several aspects of molecular biology and therapeutic options for ATC. We hope that this special issue could contribute to the advancement of the knowledge of this rare and aggressive cancer.

